# Fab on a Package: LTCC Microfluidic Devices Applied to Chemical Process Miniaturization

**DOI:** 10.3390/mi9060285

**Published:** 2018-06-05

**Authors:** Houari Cobas Gomez, Roberta Mansini Cardoso, Juliana de Novais Schianti, Adriano Marim de Oliveira, Mario Ricardo Gongora-Rubio

**Affiliations:** 1 Micromanufacturing Laboratory, Center for Bionanomanufacturing, Institute for Technological Research, 05508-901 São Paulo, Brazil; hcobas@ipt.br (H.C.G.); jschianti@gmail.com (J.d.N.S.); 2 Supramolecular Chemistry and Nanotechnology Laboratory, Department of Fundamental Chemistry, Institute of Chemistry, University of São Paulo, 05508-000 São Paulo, Brazil; roberta.cardoso@usp.br; 3 Laboratory of Chemical Processes and Particle Technology, Center for Bionanomanufacturing, Institute for Technological Research, 05508-901 São Paulo, Brazil; amarim@ipt.br

**Keywords:** microfluidic devices, LTCC technology, chemical process intensification, miniaturization, synthesis of nano and microparticles, gold nanoparticles

## Abstract

Microfluidics has brought diverse advantages to chemical processes, allowing higher control of reactions and economy of reagents and energy. Low temperature co-fired ceramics (LTCC) have additional advantages as material for fabrication of microfluidic devices, such as high compatibility with chemical reagents with typical average surface roughness of 0.3154 μm, easy scaling, and microfabrication. The conjugation of LTCC technology with microfluidics allows the development of micrometric-sized channels and reactors exploiting the advantages of fast and controlled mixing and heat transfer processes, essential for the synthesis and surface functionalization of nanoparticles. Since the chemical process area is evolving toward miniaturization and continuous flow processing, we verify that microfluidic devices based on LTCC technology have a relevant role in implementing several chemical processes. The present work reviews various LTCC microfluidic devices, developed in our laboratory, applied to chemical process miniaturization, with different geometries to implement processes such as ionic gelation, emulsification, nanoprecipitation, solvent extraction, nanoparticle synthesis and functionalization, and emulsion-diffusion/solvent extraction process. All fabricated microfluidics structures can operate in a flow range of mL/min, indicating that LTCC technology provides a means to enhance micro- and nanoparticle production yield.

## 1. Introduction

Microfluidics continuous flow techniques display advantages when compared to batch chemical processes, such as high surface-to-volume ratio, small thermal inertia, fast temperature changes, easy adjustment of residence times, and production of emulsions and suspensions with monodisperse distribution [1,2]. Microfluidic devices have been used in many applications in pharmaceutical, biotechnological, and chemical industries such as fine chemical synthesis, crystallization, and combinatorial synthesis [3,4,5].

The chemical process area uses successfully microfluidic reactors to produce micro- and nanoparticles [6]. The presence of micro- and nanoparticles within most different products such as cosmetics and pharmaceutics [7], fuel, food [8], and paints can improve product characteristics such as bioavailability of pharmaceutical ingredients [9], reduce pollutants or enhance performance of energy consumption [10], introduce nutritional ingredients in food, and so on.

A shortcoming of the latter approach is that typical microfluidic devices works in low flow ranges, such as hundreds of µL/min, preventing adequate application for industrial production purposes. Scaling-out or Numbering-up which consists of assembling several microreactors to obtain macroscopic flows maintaining the process performance can overcome this problem [11]; therefore, using structures that manage higher flow ranges is desirable.

Microfluidic reactors can be fabricated in different materials such as polymers, glasses, silicon, metals, and ceramics, using several techniques [12]. Low temperature co-fired ceramics (LTCC) green tape ceramic substrates present interesting advantages such as the possibility of microfabricating 3D geometries and can be used for higher flow rates. LTCC material is chemically inert to most solvents, it has a hydrophilic surface behavior [13], presents a low thermal coefficient of expansion, can hold out high operational temperatures, and can withstand high internal pressures, allowing implementation of several chemical process with applications in stringent environments [14,15,16]. Applications of LTCC microfluidic devices to carry out several chemical process operations are reported in the literature, including bioreactors [17,18,19], enzymatic reactors [20], combustors [21,22], microthrusters [23], mixers [24,25], chemical reactors [26,27], heat exchangers [28], and gold nanoparticles generators [29].

Nanotechnology is a branch of science with the objective of the development of materials with unique properties. Nanomaterials are composed of particles with at least one dimension sized from 1 to 100 nanometers and have different properties than their macrometric analog which guarantees great success in several areas such as optical, sensors, electronics, catalysts, energy conversion, and medicine [30]. However, there is a huge problem associated with controlling several parameters that define synthesis and other reactions of these materials, which can lead to polydispersed and low-quality nanoparticles.

Miniaturization through microfluidics permits faster, controlled, and reproducible chemical processes. Advantages such as high heat and mass transfer of reagents, low reagent and energy consumption, safety, portability of reactors, and so on, enable easier conversion of laboratory routes to larger scales once they guarantee the main control of process, in contrast to difficulties related to converting such a reaction to large-scale reactors [31].

However, microfluidic devices currently employed in the synthesis of nanomaterials have a high manufacturing cost (e.g., silicon) or are not compatible with several reagents (e.g., steel, polymer, etc.). One of the promising technologies used in microfabrication is LTCC, which consists of easy-to-process hybrid ceramics with physical-chemical properties suitable for fabrication of microreactors applied to synthesis and modification of nanoparticles. LTCC devices are designed using multiple layers of green ceramics, which will generate 3D structures after aligning them and applying heat and pressure to the lamination. After sintering, green ceramics are compatible with organic solvents, strong acids, and reducing reagents; that is, its application leads to a wide range of reactions using LTCC devices [16].

At this time, we present a review of the utilization of LTCC microfluidic devices, developed in our laboratory, applied to chemical process miniaturization, operating in the mL/min flow rates range.

LTCC microfluidic devices with different designs in a 3D configuration were fabricated in order to generate micro- and nanoparticles under continuous flows, as well to perform other unit operations. Each design and configuration took into consideration chemical process such as ionic gelation, emulsification, nanoprecipitation, solvent extraction, nanoparticle synthesis and functionalization, and emulsion-diffusion/solvent extraction.

## 2. Materials and Equipment

The next two tables explain comprehensively the materials and equipment adopted for the present work.

### 2.1. Materials

Table 1 depicts the materials used in the reported applications.

### 2.2. Equipment

Table 2 depicts the equipment used in the reported applications.

### 2.3. LTCC Microfabrication

**Figure 1:**
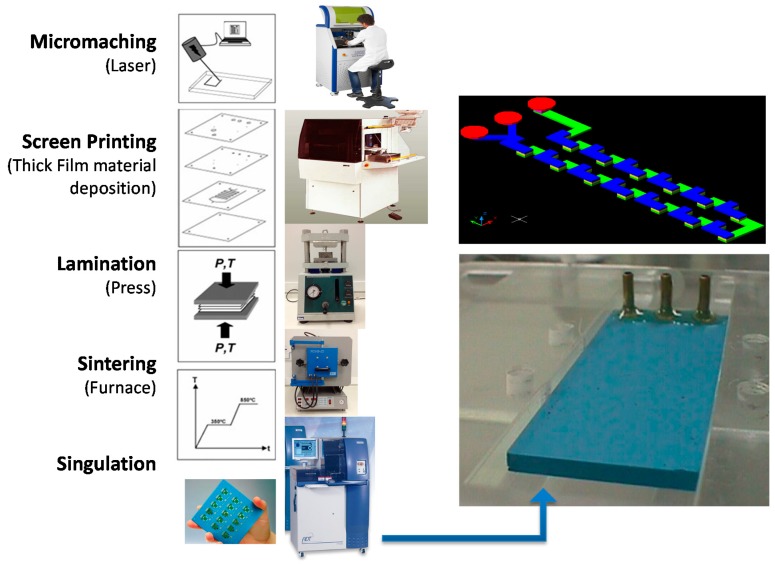
Low temperature co-fired ceramic (LTCC) process to fabricate microfluidic devices.

The microfluidic devices were fabricated employing the LTCC process as shown in Figure 1 (cutting layers, thick film deposition, lamination, sintering, and dicing) [16]. The layers with the microchannel geometries were fabricated using a diode pumped IR laser, model U-15 1064 nm Ultrafast Laser Maker (RMI Laser, LLC, Lafayette, Colo.) and a prototyping machine equipped with an ultraviolet laser (355 nm wavelength), model LPKF Protolaser^®^ U3 (LPKF Laser & Electronics AG, Garbsen, Germany). Thermo-compression lamination process is done by means of a uniaxial laminator and sequential lamination schedule using 11.8 MPa of pressure at 70 °C (hydraulic press machine, Model MA098/A30, Marconi, Piracicaba, São Paulo, Brazil). The lamination procedure lasts 20 min (after 10 min the device was rotated 90° and laminated again for another 10 min). In a sequential lamination schedule, layers with the same microchannel cutting project were laminated together forming an individual block; finally all blocks were laminated together for a final laminated device.

The team’ experiences acquired during device manufacturing lead us to use a baking stage prior to lamination. When performing a 20 min/60 °C baking prior to the lamination procedure we observed an improved final lamination process characterized for a better adhesion between green tapes. The sintering process was done in a muffle furnace (EDG Equipment, model EDG10P-S, São Paulo, Brazil), in two stage profiles: first, heating the device at a 6 °C/min ramp, with plateaus at 450 °C per 30 min and 850 °C per 30 min. In some cases, different types of sacrificial materials were used to avoid structure sagging: high purity carbon sheets (127 μm thickness, TCS-CARB-1 from Harmonics) and ceramic powder liner (Zirconia, CPL-AL-1 from Harmonics) and ESL Fugitive Tape 49000 (Electro Science Laboratories, USA). The input and output brass or Polyphenylene sulfide, PPS (Fortron) fluidic interconnection tubes were glued to the ceramics using a high temperature epoxy (EPO-TEK 353ND, Epoxy Technologies, Billerica, MA, USA). The gluing process was performed by means of a hot plate at 150 °C. Other microfluidic devices use a microfluidic connector that support high pressures and flow rates in a fluidic line.

Fired glass-ceramics display good resistance to several aggressive wet chemicals. On the other hand, certain chemicals etch the glass-ceramic material, preventing their use in microreactors. Chemical resistance of LTCC materials was studied in [32] aiming for its use in harsh chemical processes applications. Sometimes microreactors utilize high temperatures to implement a chemical procedure. LTCC can work properly in temperatures up to 400 °C as investigated in [33].

Occasionally local porosity obtained by etching is used to render certain substrate modifications. A process to locally generate a defined porosity in LTCC in the fired state using phosphoric acid etching for dielectric properties modulation is described in [34].

We developed different geometries such as vortex, 3D serpentine, and coaxial flow focusing on 3D for chemical process implementation as described hereafter.

## 3. Microfluidic Devices for Chemical Process Implementation

### 3.1. LTCC 3D Flow Focusing Device

**Figure 2:**
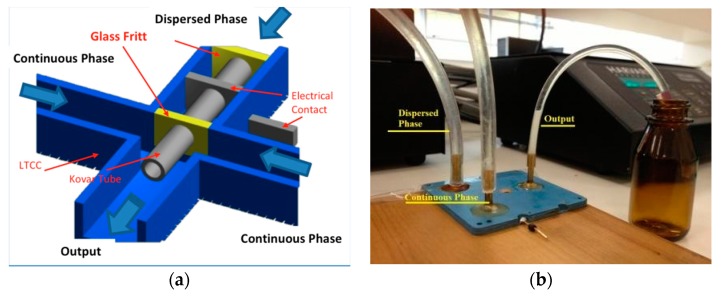
Coaxial flow focusing device: (**a**) cross section with Kovar tube to allow coaxial structuring and (**b**) the complete Flow Focusing Device in 3D configuration (3D-FFD) in operation.

The first device design used was a Flow Focusing Device in 3D configuration (3D-FFD) presented in Figure 2a [35,36]. In the device cross junction, a Kovar tube is inserted to obtain a 3D structure. The Kovar tube has an inner diameter of 500 µm; it is inserted manually before the sintering process. The complete system is shown in Figure 2b. The device also displays an electrical contact connected to the metal tube in order to modify particle diameter with electric potential [37]. In the present paper, the electrical contacts were not used for the reported experiments.

This design is useful for produce emulsions and also to synthetize nanoparticles. A dispersed phase is introduced inside a central channel and is focused by a continuous phase by side channels. In order to produce a single emulsion, two immiscible fluids, usually oil and water, are introduced in this structure [38,39,40]. Depending on the fluids properties and fluid flow rate, a droplet could be performed in a highly homogeneous way. In order to obtain nanoparticles, a crystalized solid material is diluted in a solvent and introduced inside the device as a dispersed phase. An aqueous phase is used as continuous phase and under flow rate control it is possible to focus the dispersed phase.

Depending on the flow rate ratio, the dispersed phase flows as a tiny flow surrounded by water. This environment promotes the solvent diffusion to water, allowing the crystalized material to nucleate and form a nanoparticle.

The flow rate and ratio between phases are the keys to control the droplet and nanoparticle size [29,35,36]. As a first application, the 3D-FFD device was applied to produce sodium alginate microparticles by an ionic gelation process [41,42,43]. The ionic gelation is a process to obtain polymeric microcapsules. An ionic polymer, sodium alginate or chitosan for example, interacts with a fluid with opposite charge promoting a cross linking. One way to obtain this process is called external ionic gelation. This process begins with the ionic polymer dispersed in a single emulsion, and on sequence, the droplets reach an ionic liquid. The formed capsules have been widely used in controlled released, to encapsulate living cells, vitamins, proteins and so on. Sodium alginate is a biopolymer widely used in the ionic gelation process and was used to obtain microcapsules in the 3D-FFD.

The same device (3D-FFD) was used for nanoprecipitation of Benzophenone-3 (BZ-3), a typical sunscreen agent used in cosmetic products [37,44,45]. As mentioned previously, nanoprecipitation processes occur after a solvent diffusion across the water. The BZ-3 was diluted in two different solvents: Isopropyl Alcohol (IPA) and Methanol (MTH). The influence of these solvents in the particle sizes were observed. We used the BZ-3 concentration value of 7.5 mg/mL. The influence of total fluid flow rate and the ratio between fluid flows (*R* = *Q*_water_/*Q*_B-3_) over particle sizes was also observed. The BZ-3 and water are injected into the device using the dispersed and continuous phases inputs respectively.

#### 3.1.1. Sodium Alginate Microparticles

Sodium alginate (1.5% *v*/*v*) was diluted in ultra-pure water and introduced in microfluidic channels as dispersed aqueous phase to obtain sodium alginate microcapsules. Mineral Oil was used as continuous phase.

As consequence of 3D-FFD microfluidic device operation, water in oil emulsion with highly homogeneous droplets size was produced. Syringe pumps control both fluids. This emulsion was immersed in a calcium chloride solution (4% *v*/*v*), where the sodium alginate droplets react with calcium ions to form a gel.

The continuous phase flow rate was varied from 200 μL/min to 3 mL/min. The ratio of continuous phase and dispersed phase, *R* = *Q*_c_/*Q*_d_, was varied from 10 to 100. The microcapsules were evaluated by digital microscope VHX-6000 (Keyence, Osaka, Japan).

**Figure 3:**
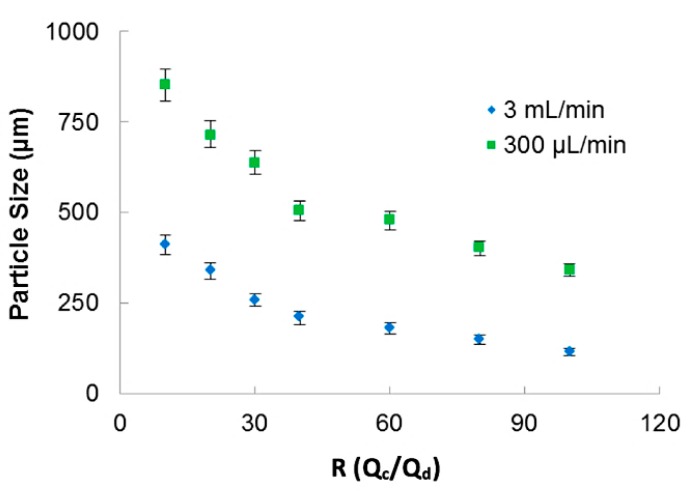
Sodium alginate particle size as a function of the ratio between phases for two different continuous flow rate.

In Figure 3, the relation of particle sizes versus the ratio between the phases for two applied total fluid flow rates is presented. The 3D-FFD device presented smaller particles in higher continuous phase flow rate (3 mL/min). In this case, particles ranging from 400 µm to 115 µm with polydispersity index less than 1.5% (PDI = (σ/d)^2^) were obtained. Polydispersity index (PDI) is defined as the square of the standard deviation/mean diameter. It was observed that when flow ratio between the phases is increased, particle size became smaller. This behavior is expected for most microfluidic devices applied for emulsion production. The emulsion droplets became smaller in size when the continuous flow rate is higher. During droplet formation, just small amounts of dispersed phase can reach the continuous phase [46].

**Figure 4:**
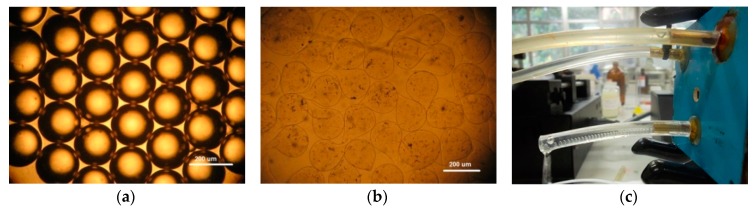
Sodium alginate microparticles: (**a**) sodium alginate droplets before ionic gelation, (**b**) sodium alginate microparticles after ionic gelation, and (**c**) the 3D coaxial device in operation. White bar scale of 200 µm.

In Figure 4a is shown a monodisperse emulsion of sodium alginate droplets without gelation. Considering a continuous oil phase of 300 μL/min, it was possible to obtain droplets with 200 µm. After the ionic gelation process, it was possible to obtain sodium alginate microcapsules that are shown in Figure 4b. As the emulsification process inside the channels is fast, the droplets reach the ionic solution and solidify rapidly, forming microcapsules with some deformation of their shapes. Experimental arrangement of the microfluidic device used to produce sodium alginate microparticles is depicted in Figure 4c.

It is important to note that the geometry used to make the 3D coaxial structure allow the production of water-in-oil emulsions without surface treatment.

#### 3.1.2. Benzophenone-3 (BZ-3) Nanoprecipitation

Benzophenone was diluted in two solvents: Isopropyl Alcohol and Methanol in order to process Benzophenone in nanoparticles synthesis. The Benzophenone concentration used was 7.5 mg/mL, defined in previous experiments. Ultra-pure water was used as anti-solvent in this process as a continuous phase. Again, syringe pumps controlled both fluids. Particles were evaluated by DLS equipment.

**Figure 5:**
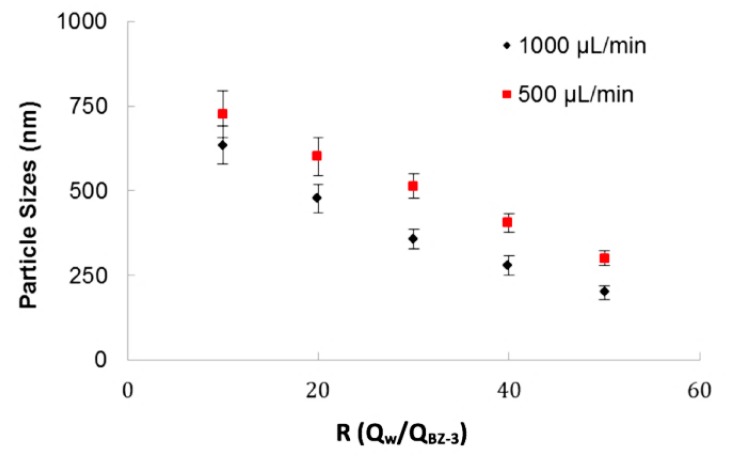
Benzophenone-3 particle size as a function of the ratio between fluid phases (water/BZ-3) for different total flow rates.

The 3D-FFD device was also used to produce particles by nanoprecipitation process. Figure 5 shows the particle size relation with ratio between fluid flows (*R* = *Q*_water_/*Q*_B-3_) for two water flow rates (*Q*_water_) of 1000 µL/min and 500 µL/min. This ratio (*R*) indicates how many times the continuous phase flow is higher than the dispersed phase. The higher the term *R* is, the smaller the droplet size gets.

It was observed that total flow rate influences the final particle size. In this example, it was verified that for a flow rate of 500 µL/min the particle sizes are higher than for 1000 µL/min. As observed on the sodium alginate experiments, this device offer better results in higher flows. It was considered that, when the devices operate in lower flows, the particles have conditions to produce coalescence. In the BZ-3 experiments, surfactants of any kind were not used. The ratio between phases, *R*, is an important parameter and for higher values of *R*, it was possible to obtain particles with smaller sizes.

**Figure 6:**
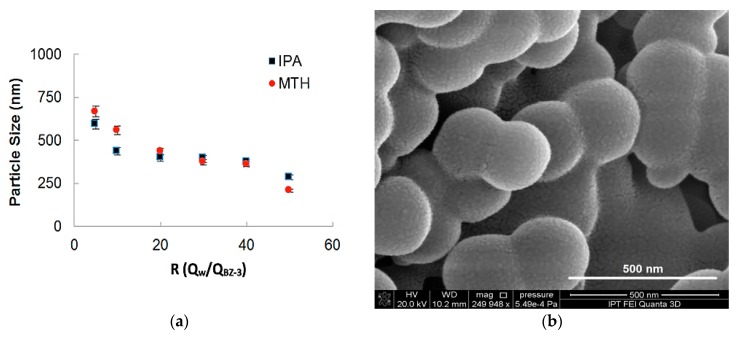
(**a**) Benzophenone-3 particle size as a function of the ratio between phases for two different solvents (isopropyl alcohol and methanol), (**b**) SEM-FEG micrograph obtained with a flow rate of 500 µL/min and BZ-3 concentration of 7.5 mg/mL, yielding mean particle size of 250 nm.

In Figure 6a the influence of the ratio between flows on the particle sizes for two different solvents (Methanol and Isopropyl Alcohol) is shown. The total flow rate for both experiments was maintained in 500 µL/min and the BZ-3 initial concentration was of 7.5 mg/mL. It can be observed that for higher values of *R* is possible to obtain smaller particles. BZ-3 particles were obtained in a range varying from 210 nm up to 850 nm and the size differences obtained for IPA and MTH was not so high. As MTH has more toxicity, so the use of IPA presents a better strategy for nanoprecipitation for this pharmaceutical ingredient. We show BZ-3 morphology in a SEM-FEG micrograph presented in Figure 6b.

### 3.2. Vortex Micromixer for Water-in-Diesel Emulsion

A vortex micromixer was used to produce a water-in-diesel emulsion [47,48]. The introduction of water in diesel fuel, as an emulsion, is one of the attempts to reduce the emission of pollutants [49,50]. Usually, some commercial water-in-diesel emulsions contain droplets with sizes from 3 to 10 µm. Water-in-diesel emulsions present advantages such as reduced motor power consumption and lower temperature. The presence of water nanodroplets in diesel causes micro explosions in the combustion process. This microexplosions increase the presence of oxygen, resulting in an enhanced fuel performance. It was also demonstrated that small amounts of water nanodroplets (5 to 14%) could reduce the NO, NOx, and CO_2_ emission in a range of 18% [10,51,52,53,54]. The micromixers are helpful to obtain emulsions in a continuous way and are presented as a tool to produce water-in-diesel emulsion inside an engine. Vortex geometry has been used for intensifying mixtures by promoting vortices between fluids, and it was applied to produce a simple emulsion. We analyzed the influence on the sizes of water particles by changing parameters, such as surfactant concentration and total flow rate, maintaining the ratio between water and diesel phases.

In these experiments, ultra-pure water was mixed with Ethanol and introduced in the device as dispersed phase in a proportion of 5:1, with two different surfactant concentrations (5% and 10% *v*/*v*). The surfactant used for the experiments was Nonyphenol-Ethoxylated, Renex18:Renex100 (1:1). The diesel is introduced as continuous phase, with flow rates varying from 8 to 16 mL/min. Syringe pumps were used to supply the fluids. We analyzed the water droplets’ size observing parameters such as surfactant concentration and total fluid flow of continuous flow. In all experiments, 5% of water flow to 95% of diesel flow (5:95) was used. Water-in-diesel emulsion was evaluated by DLS equipment.

**Figure 7:**
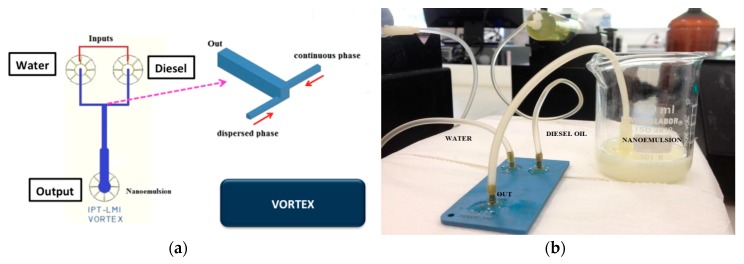
Vortex microfluidic device: (**a**) device geometry; (**b**) fabricated device in LTCC.

A vortex micromixer (Figure 7a) was used to produce water-in-diesel emulsion. Water and oil flows inside the vortex structure into the not aligned input channels and the fluids are mixed by vortices promoted by high values of flow rate compared to common microfluidic devices. In this geometry, two side channels introduce fluids simultaneously in a bigger channel promoting vortex formation. The vortex channel has a hydraulic diameter of 1.7 mm and the side channels have a hydraulic diameter of 700 µm in size. Figure 7b shows the LTCC vortex device in operation for water-in-diesel emulsion.

**Figure 8:**
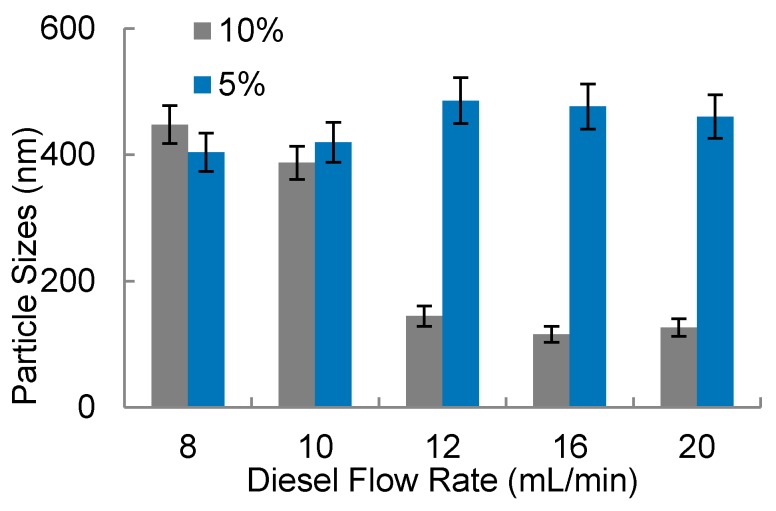
Water-in-diesel emulsion: water droplets size in function of diesel flow rate for two surfactant concentration (5% and 10% *v*/*v*).

As considered before on the 3D-FFD, the water droplets’ sizes were evaluated observing the influence of flow rates. In Figure 8 the water droplets’ sizes (in nanometers), in a function of diesel flow rate, is shown.

**Figure 9:**
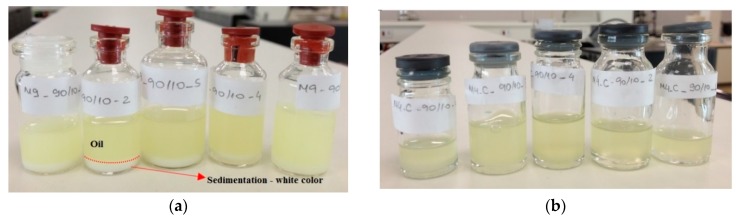
Water-in-diesel nanoemulsion: (**a**) fuel with 5% of surfactant, which it is possible to observe a white sedimentation and (**b**) fuel with 10% of surfactant with a translucent aspect.

It was observed in Figure 8 that the smallest amounts of surfactant (5% *v*/*v*) present droplets with higher sizes compared to the experiments that used 10% surfactant. Additionally, with 5% of surfactant, water droplets present sizes between 400 nm up to 600 nm with poor stability, presenting a milky aspect, promoting phase separation after some minutes. In higher flow rates combined with higher surfactant concentration (10%), it was possible to note water droplets with smaller sizes, in a range below 200 nm. The diesel flow rate with this surfactant condition (10% *v*/*v*) presents more influence in the water particle sizes. In Figure 9a final solutions obtained with 5% of surfactant are shown. In this picture, it is possible to observe a white region which indicates a phase separation. This indicates that water nanodroplets coalesced and separated from the oil. The white sediment is the surfactant with water. In Figure 9b, upon inspection, it can be seen that the solution is translucent and homogeneous. Usually the homogeneity of color solution indicates more stability. The particle size measurements were also helpful to analyze the solution stability.

**Figure 10:**
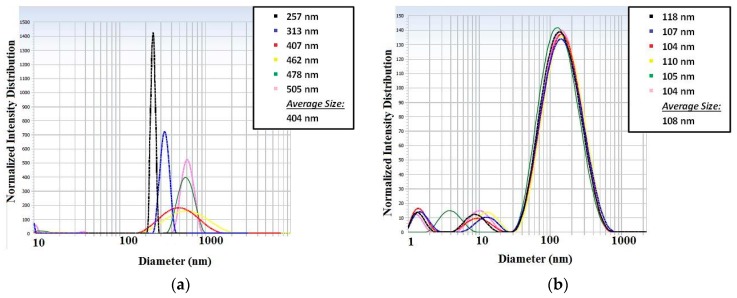
Particle sizes measurements: (**a**) results obtained for a solution with 5% of surfactant and (**b**) results obtained for a solution with 10% of surfactant.

In the case with low surfactant concentration (5%) it was possible to see that the water droplets sizes were increasing after each measurement on the picture shown at Figure 10a. On the other hand, observing the particle size measurement of the high surfactant concentration, in Figure 10b, it is possible to see a stable in time solution, maintaining the droplets’ size values in the same range. The curves are presented in different colors for different measurements of the same sample. The legend indicates the average droplet size obtained.

It was observed that microfluidic devices can offer water droplets in nanoscale range. The sizes depend on flow rate and stability varies with the surfactant concentration. It is still a challenge to find a surfactant combination that can offer a strongly stable nanoemulsion. However, it is important to highlight that stability is not crucial for fuel use, as the concept is to introduce the microfluidic device inside an automotive engine, to prepare a water in diesel emulsion in situ. In this way, the next step for this work will be to introduce the vortex device in a motor to evaluate the efficiency and pollutants emission obtained with the modified fuel.

### 3.3. LTCC 3D Flow Focalization Device for Liquid-Liquid Partial Solvent Extraction

This 3D flow focalization microfluidic device is intended for a partial solvent extraction scheme. The proposed system aims to partially extract the solvent present in a mixture containing aqueous and organic phases. Liquid-liquid extraction with microfluidic devices is based on contacting two different liquids flowing through a microchannel. At the contacting interface a diffusion gradient is established allowing the material diffusion from the highest concentration liquid to the lower one. Different microfluidic approaches have been used, as an example H channels configuration [55] and droplet based liquid-liquid extraction devices [56,57] can be mentioned. Hydrodynamic flow focalization could be used in order to improve the diffusion process due to the increase in the fluid-fluid cross section area ratio. In particular, 3D flow focalization has been shown to be an interesting alternative for the diffusion process improvement [36,37,58,59,60]. Unfortunately, 3D microfluidics devices already reported in the scientific literature do not allow solvent extraction because devices have only one output designed for mixing purposes.

**Figure 11:**
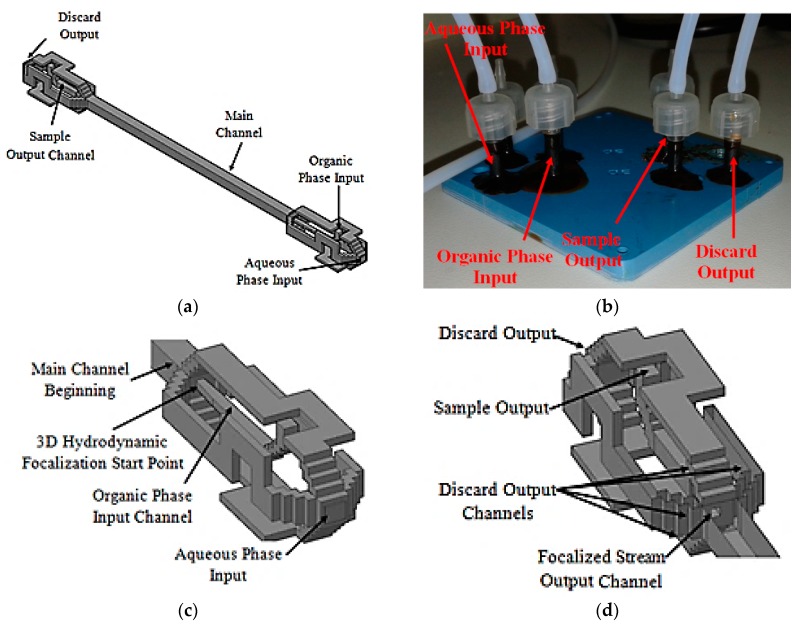
Microfluidic device. Tridimensional project: (**a**) Full device; (**b**) Fabricated device; (**c**) Input channels section details; (**d**) Output channels section details.

This scheme uses a 3D flow focalization, in a partial solvent extraction task, in order to improve the organic phase (inner) diffusion to the external aqueous phase. The device is composed of three different parts, the input distribution channels, the main channel, and the output distribution channels. The designed input distribution channels ensure a centered 3D focalized solvent stream along the main channel. The focalized solvent mixes with the surrounding aqueous phase thanks to diffusion. Wisely projected output channels take the central fluid out separately from the surroundings. Thus, the device has two different outputs, one for the focalized fluid and another one for the waste fluid, which is the aqueous phase plus solvent. The proposed microfluidic device tridimensional project is shown in Figure 11a, and the fabricated device is shown in Figure 11b.

3D hydrodynamic organic phase focalization along the main channel is assured thanks to the projected input channels configuration [58], Figure 11c. The main channel was designed with a length of 21.4 mm and a hydraulic diameter of 772 µm. The projected output channels configuration is similar to that used at the input stage, Figure 11d. The main difference lies in shorter discard output channels and sample output channel hydraulic diameter. The focalized stream output channel is centered relative to the main channel cross section and was projected with a hydraulic diameter of 214.63 µm.

Along the main channel, thanks to 3D hydrodynamic flow focalization and fluid motion, a conical-like diffusion pattern is expected. As far as fluids travel by the main channel, solvent diffuses radially from the main channel center to the walls. As a consequence, a solvent spread out at the main channel cross section output is expected. A wisely located output channel collects the focalized stream preferentially. The diffused solvent in water flows out the device by the discard outputs channels. In this way the focalized stream partially reduces its solvent content.

For a device concept proof, Acetone and deionized water were used as organic and aqueous phases, respectively.

The study was conducted in two stages. First, simulations for device microfluidics and chemical transport analysis, using COMSOL^®^ Multiphysics (COMSOL 4.2, COMSOL Inc., Stockholm, Sweden), were done with five device configurations. In the second stage, experiments were done with the device which showed better simulation results. The extraction efficiency was the variable used as an indicator for device performance validation. A factorial experimental planning with the central point [61] was used to access the effect of process variables on the extraction efficiency. For simulations, those variables were flow rate ratio (*R*_Q_), total flow rate (*Q*_T_), main channel length (*L*_CD_), and focalized stream channel output hydraulic diameter (*OD*_H_). By its time, physical experiments used *R*_Q_ and *Q*_T_ as process variables. Experiments were conducted in triplicate for statistical analysis. Statistica13^®^ software (StatSoft Inc., Tulsa, OK, USA) was used for analyzing simulation and experimental results. Used software calculates automatically the Pareto chart and the predicted extraction efficiency mean value. Table 3 summarizes the variables values and conditions used.

Flow ratio is defined by the following relationship:

(1) $$R_{Q}=\frac{Q_{A}}{Q_{O}}$$

were, $Q_{A}$: Aqueous phase flow rate, $Q_{O}$: Organic phase flow rate.

Then Total flow rate is:

(2) $$Q_{T}=Q_{A}+Q_{O}$$

Main channel length is defined as in Figure 11a

Output hydraulic diameter is defined by the following relationship:

(3) $$OD_{H}=\frac{2\times \left(a\times b\right)}{a+b}$$

were, *a*: Focalized stream output channel width; *b*: Focalized stream output channel height

The extraction efficiency was calculated as in (4).

(4) $$\eta \left(\%\right)=100\times \frac{V_{aceD}}{V_{aceD}+V_{aceS}}$$

where, *η* is Extraction efficiency in %, *V_aceD_* is Acetone volume at discard output, *V_aceS_* is Acetone volume at sample output.

The *V_aceD_* and *V_aceS_* values were calculated as in (5).

(5) $$V_{ace}=\frac{C\times V\times MM}{\rho }$$

where, *C* is Acetone concentration (mol/m^3^), *V* is Collected volume at the sample or discard outputs (m^3^), *MM* is acetone molar mass (58.079 g/mol), *ρ* is acetone density (791,000 g/m^3^).

The acetone concentration was determined at the output boundaries for simulation experiences or experimentally by means of gas chromatograph.

**Figure 12:**
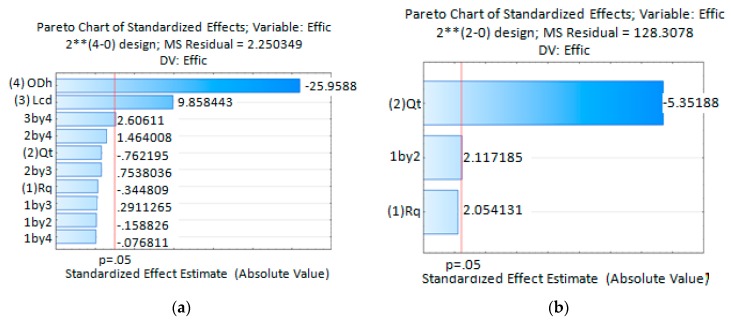
Statistical analysis results, Pareto Chart of Standardized Effects for the extraction efficiency (**a**) For simulation experiences; (**b**) For experiments done with microfluidic device with *OD*_H_ = 214.63 µm and *L*_CD_ = 21.4 mm.

The Pareto Chart of Standardized Effects for the extraction efficiency (Effic) obtained from simulation results, Figure 12a, indicates that extraction efficiency is highly influenced by the process variables *OD*_H_, *L*_CD_ and the interaction between them. The extraction efficiency is inversely proportional to *OD*_H_ and directly proportional to *L*_CD_ and the interaction between them. As the *OD*_H_ decreases, the diffused solvent portion it takes out from the device is lower and hence the major solvent portion is extracted via the discard output channel. As a consequence, the extraction efficiency increases. For longer *L*_CD_, the diffused solvent attains a wider distribution in the main channel cross section. As the sample channel output is located at the main channel center a reduced amount of solvent leaves the device by the sample channel output, increasing this way the extraction efficiency. Simulate data analysis done using Statistica13 predicts extraction efficiency mean values better than 53.8% for the process variables *OD*_H_ and *L*_CD_. The best configuration was obtained for the lowest *OD*_H_ and highest *L*_CD_ values. In this case the predicted extraction efficiency mean value was 80.6%. Those values were obtained for all combinations used for the process values *R*_Q_ and *Q*_T_.

For the experimental stage, a device with *OD*_H_ = 214.63 µm and *L*_CD_ = 21.4 mm was fabricated. Statistical analysis obtained from experimental data shows the *Q*_T_ as the statistically significant process variable influencing the extraction efficiency, Figure 12b Pareto Chart of Standardized Effects for the extraction efficiency obtained experimentally. The extraction efficiency is inversely proportional to the variable *Q*_T_ and directly proportional to the interaction between variables *Q*_T_ and R_Q_ which is also shown to be statistically significant. Taking this into account, this device will offer higher extraction efficiency when working with lower *Q*_T_. In this condition the fluid will take a longer time to reach the outputs due to the fluid mean velocity reduction. This increase in the residence time allows a wider uniform distribution of solvent at the main channel output. As a consequence, the amount of solvent leaving the device by the sample output channel is reduced and the extraction efficiency increased. The higher statistically predicted mean values, 77% and 76.6%, were obtained for the lower *Q*_T_. Those values are in agreement with simulation results. The minimum efficiency predicted mean value of 28.2% was also obtained, which does not agree with values obtained by simulations. Avoiding the process variables combination where *Q*_T_ = 10 mL/min and *R*_Q_ = 5, it can be said that with such a device it is possible to predict statistical mean extraction efficiencies higher than 55.5%.

**Figure 13:**
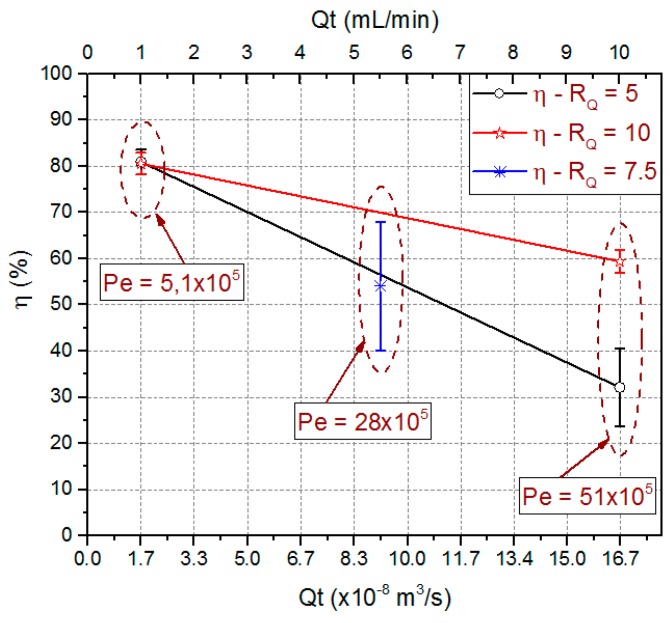
Predicted means values for extraction efficiency and Péclet number for the process variables *R*_Q_ and *Q*_T_. Experimental values obtained for microfluidic device with *OD*_H_ = 214.63 µm and *L*_CD_ = 21.4 mm.

Experimentally obtained extraction efficiency data are also presented in Figure 13. This figure shows the extraction efficiency mean value, the standard deviation from triplicate data, and the Péclet number (*P_e_*) for used combinations. The obtained *P_e_* number values were 5.1 × 10^5^, 28 × 10^5^ and 51 × 10^5^ for total flow rate of 1 mL/min, 5.5 mL/min and 10 mL/min respectively.

(6) $$P_{e}=\frac{L\times v}{D}$$

were, *L* is characteristic length, *v* is mean flow velocity, and *D* is Diffusivity coefficient.

The *P_e_* number is a dimensionless number used in the mass transport phenomenon studies in fluids. In chemical species mixing it is helpful for determining when a mass transport is mainly due to convection or diffusion. It relates convection to diffusion transport mechanisms as in (6).

Those results show Convection as the predominant mass transport mechanism. For 1 mL/min, *P_e_* number is an order of magnitude lower than for 10 mL/min. This is an indication that a diffusive flow component is higher for the lower total flow rate, justifying an increase in the extraction efficiency for the same flow rate ratio. Extraction efficiencies in the order of 80.8% ± 2.2% were obtained for *Q*_T_ = 1 mL/min independently of the used *R*_Q_. For higher *Q*_T_ values, the best results were obtained for the higher *R*_Q_ values. A way to decrease the *P_e_* value and hence to increase the extraction efficiency would be increasing the diffusivity coefficient which could be done increasing fluid temperatures.

### 3.4. LTCC Microfluidic Devices Applied on Synthesis and Functionalization of Gold Nanoparticles

The Turkevich Method is one of the most known routes for synthesis of nanoparticles: the original route consisted of synthesis of spherical gold nanoparticles stabilized with citrate [62]. This route requires only two reagents: sodium citrate/citric acid and gold (III) salt-citrate plays the role as a reducing agent of gold (III) and passivation agent of nanoparticles, avoiding extra agents to stabilize them. This route guarantees that the final product is a water-dispersible, biocompatible, and reactive nanoparticle, so they are excellent for modification and application on posterior biological studies [63].

This method requires high control of several factors simultaneously: parameters such as pH [64], concentration, and order of addition of reagents [65] influence particle growth kinetics and alter the final result. Therefore, there are many advantages on using microfluidic devices for synthesis of gold nanoparticles, once they allow production of nanomaterials with high reliability and reproducibility [66].

However this method allows synthesis of nanoparticles of different sizes and shapes, such as spherical, hexagonal [67], or nanorods [68]. In addition, the literature contains several examples of Turkevich gold nanoparticles used for development of reactions that require high control to lead to complex particles such as Janus nanoparticles [69,70]. This method becomes more valuable for adaptation from batch conditions to continuous flow and impels the development of devices that work in series in order to produce a single apparatus for synthesis, functionalization, and/or purification. Another advantage is that synthesis is performed using solutions at higher concentrations as compared to batch fabrication, where the reaction is difficult to control, and easily lead to formation of aggregates.

One of the biggest problems associated with the Turkevich Method is the need to heat the solutions near 100 °C in order to activate reducing power of citrate. It is found in literature examples of glass capillaries heated on hot plate [71], or application of microwaves in LTCC micrometric channels [72], so it was desirable to construct a device adapted for all peculiarities of this kind, containing high residence time for heating the reagents separately, and connected to a serpentine mixer for controlled mixing when both are already heated.

**Figure 14:**
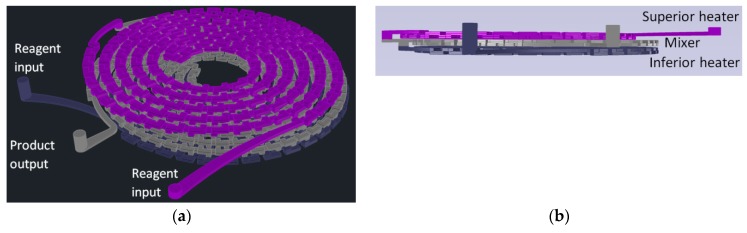

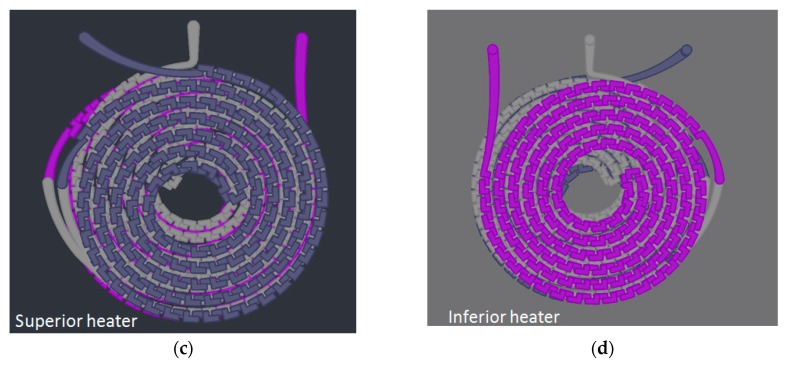
Design of a reactor for synthesis of gold nanoparticles by the Turkevich method. (**a**) Perspective view; (**b**) Frontal View; (**c**) Top view; (**d**) Bottom view.

**Figure 15:**
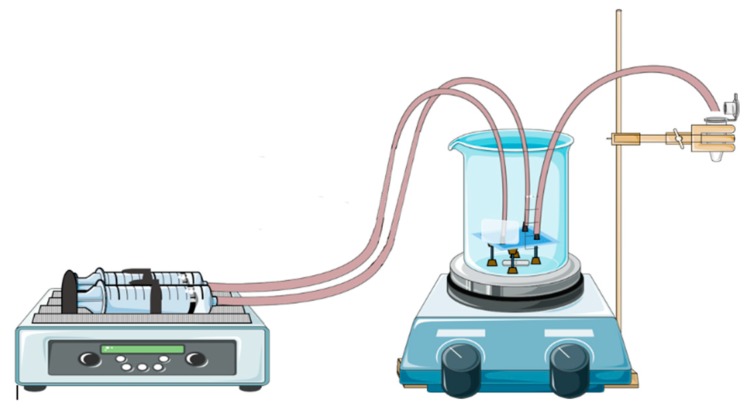
Schematization of operation of LTCC device applied on synthesis of gold nanoparticles and the heating system based on boiling-water bath.

For gold nanoparticles synthesis, our LTCC microfluidic device contains 21 951PX ceramic sheets. The manufactured device consists of three sectors: two responsible for previous heating of the reagents on separated channels (upper and lower stages), and one for subsequent mixing (intermediate stage) which acts as a controlled mixer of reagents and generator of nanoparticles. A passive mixer is made of L-segments channels that allow controlled mixing under laminar flow and was constructed with quadrilateral channels with average width and height of 350 and 500 μm, see Figure 14. The circuit is meandered in order to increase the structural resistance of the device during its manufacture. After cutting, ceramic layers were aligned in order to form a 3D structure, pressed and sintered. All connections and hoses are compatible with the reagents used in the Turkevich method. The microreactor was heated by a water bath (as seen in Figure 15). The reaction was conducted with 5 mM chloroauric solution and variable-concentration sodium citrate solution. DLS estimates the diameter of the synthesized nanoparticles, based on their hydrodynamic radius. TXRF measurements were performed in order to control gold concentration of reagents and to monitor gold concentration of the product, also to check for leaks or precipitation of gold inside the device. The obtained electronic spectrum of the nanoparticles monitors the final products.

**Figure 16:**
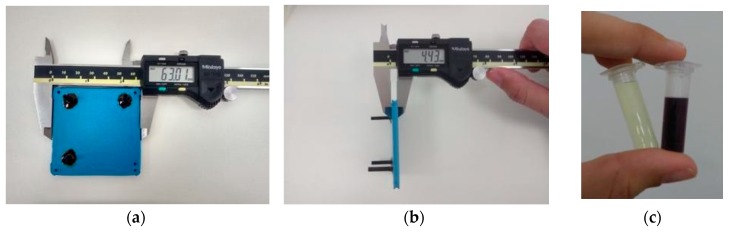
(**a**,**b**) Microfluidic device fabricated with LTCC technology employed on synthesis of gold nanoparticles; (**c**) Gold (III) salt used in nanoparticles synthesis (left) and gold nanoparticles obtained through flow conditions (right).

The final LTCC device has a thickness of 4.43 mm, as shown in Figure 16. The total volume of the device is 1.2 mL, generating a LTCC device for controlled synthesis of gold nanoparticles and sufficient residence time to heat and mix reagents. The reaction time was reduced from 30 min (usual batch conditions) to about 1.5 min. High concentrated gold solutions are used as starting reagents—usual concentrations of batch reactions are 10× diluted, as are the concentrations of sodium citrate solutions. In addition, microfluidics devices enable economy of reagents and energy, once each condition of the experiment is studied by generating only the amount of particles necessary for its characterization. On the other hand, batch conditions consist of protocols which do not allow drastic changes in reagent volumes.

The LTCC device is shown to be resistant to strong acids and organic solvents. Thus, it is easily used with other solutions once it is cleaned by Aqua Regia. A water bath was used for heating during performance due to its practicality since it maintains a necessary temperature for the reaction event, but other forms of heating bath may have proved feasible, such as glass beads, sand, or silicone bath.

**Figure 17:**
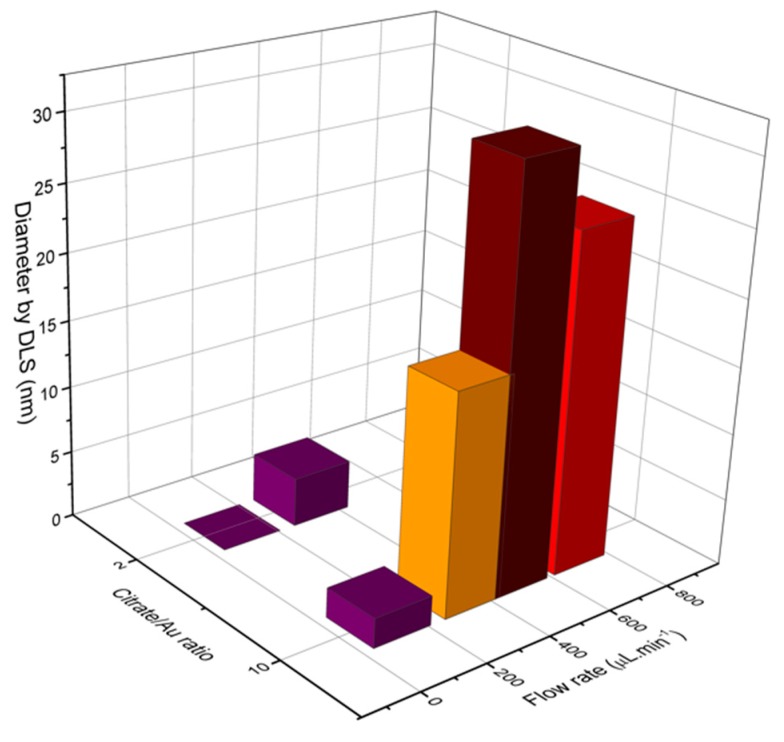
DLS measurements of gold nanoparticles obtained through the main operating conditions of LTCC device.

**Figure 18:**
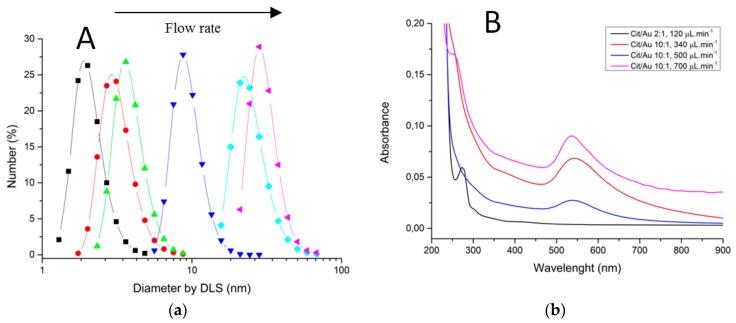
Characterization of gold nanoparticles obtained through LTCC devices. (**a**) Dynamic Light Scattering. (**b**) Electronic Spectroscopy.

Characterizations of fabricated materials by DLS and UVVis are shown on Figure 17 and Figure 18. It was possible to obtain gold nanoparticles of various diameters by modulating operation parameters of the fabricated microfluidic device (Figure 17 shows influence of reagent concentration and flow rate on hydrodynamic diameter of particle). The studied flow rates were 0.1 mL/min and 0.7 mL/min. DLS measurements indicate that the increasing flow rate leads to larger-diameter nanoparticles (Figure 18a), using 10:1 citrate/gold ratio. Electronic spectra indicate that operation at low flow rates or low citrate/gold ratios are not sufficient to complete the reaction, since they indicate the presence of remaining Au (III) ions at approximately 250–300 nm. Higher flow rates accentuate the Plasmon band located at about 520 nm (Figure 18b), typical of spherical nanoparticles at the sizes shown. Higher citrate/gold ratio also increases particle diameter. A ratio of 2:1 leads to formation of smaller particles, so this condition was poorly explored since the main goal is maximizing particle size. It is explained by the lower concentration of reducing agent in the solution, which prevents the reduction of gold and consequently particle growth. But flow rate increases the diameter of nanoparticles because it enhances the mixing effect of reagents due the device geometry.

Synthesis under flow conditions showed itself as controlled and reproducible, generating the same results even operated on different days. The main advantage of conversion of this method to continuous flow conditions is that it enables modulation of the operation, allowing the synthesis of nanomaterials with different properties (e.g., shapes and sizes), only by making small changes or adaptations in operational parameters or input reagents.

### 3.5. Continuous Regime Microfluidic System for Nanocapsules Generation

In the pharmaceutical industry, pharmaceutical active compounds are encapsulated using nanocapsules. This practice offers several advantages, including use in sustained release systems, and improvement in bioavailability and biocompatibility [73,74,75,76,77].

The emulsification-diffusion and solvent extraction/evaporation is a chemical process for encapsulation used in the pharmaceutical field, making it possible to generate particles with sizes ranging from micrometer to nanometer [73,76,78].

A traditional emulsification process requires high energy investment, as shear forces, for disperse phase drop size reduction and also to obtain a higher system stability [79]. Such a process presents a high polydispersity index and has a poor control over the particle size. Thus, it is important to develop alternative processes that make nanocapsule generation, with size control and narrow size distribution, possible. This system aims for nanocapsule generation of pharmaceutical active compounds. This is achieved by miniaturizing the emulsification-diffusion and solvent extraction/evaporation chemical process. The generated nanocapsules size setting is controlled by process variables like total flow rate through devices. It also aims for the production of nanocapsules for the pharmaceutical industry. For this, the system should be able to work at flow rates higher than that currently reported in the literature, which is approximately 40 mL/min for emulsion generation with micromixers [80,81,82,83,84,85,86,87,88,89].

**Figure 19:**
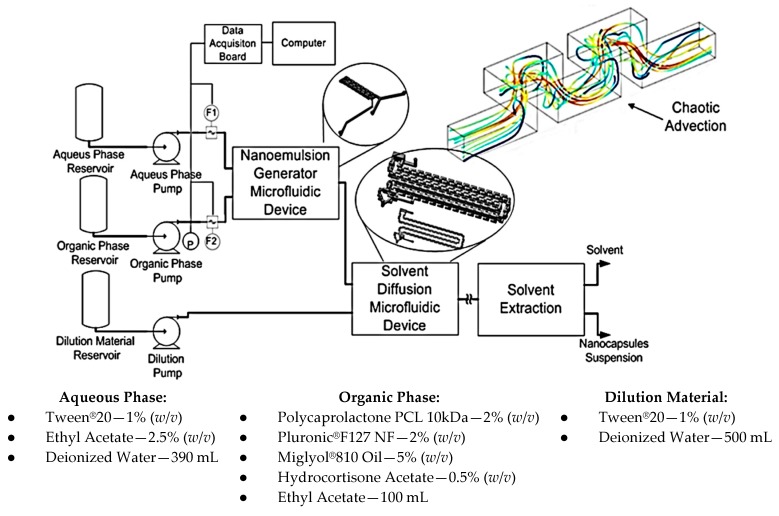
Layout for generation of nanocapsules through a continuous regime microfluidic system.

The chemical process and its steps for conventional emulsification-diffusion and solvent extraction/evaporation were analyzed. As a result, steps referring to the nanoemulsion and diffusion were considered for miniaturization through microfluidic techniques maintaining the same operation principles. Figure 19 presents the developed system.

The base microfluidic geometry selected for the emulsion and diffusion steps was the 3D Serpentine, Figure 19. This geometry executes a similar action to the mechanical agitation, enabling emulsion generation inside the microchannels when immiscible fluids are used at the entrance. 

**Figure 20:**
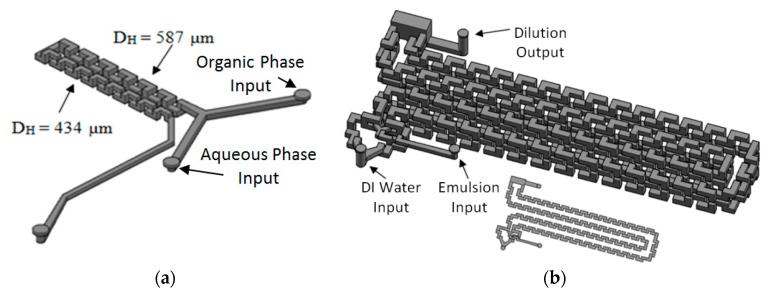
Microfluidic devices used for: (**a**) Emulsification module; (**b**) Diffusion module.

The system has three main modules. The first one generates the emulsion and is comprised of two reservoirs for aqueous and organic phases, two HPLC pumps, two flow rate sensors, a pressure sensor and the 3D Serpentine micromixer, with two sections of different hydraulic diameters (587 µm and 434 µm), Figure 20a. The second module uses another microfluidic geometry based in a 3D serpentine for solvent diffusion from inside the drop, Figure 20b. This module also has a reservoir for deionized water and a pump. Finally, there is the solvent extraction module.

Figure 19 also shows the materials used for samples preparation. The mixing of chemical components was made outside the device through magnetic stirring until phases become completely translucent. Temperature for aqueous phase and dilution was 21 °C and temperature for the organic phase was 60 °C.

For system validation, in the emulsion generation module the total flow rate swept from 10 mL/min to 65 mL/min with 5 mL/min steps while the flow rate ratio between aqueous and organic phases was four. The flow rate ratio between dilution flow rate and emulsion flow rate was kept constant at 3.5.

**Figure 21:**
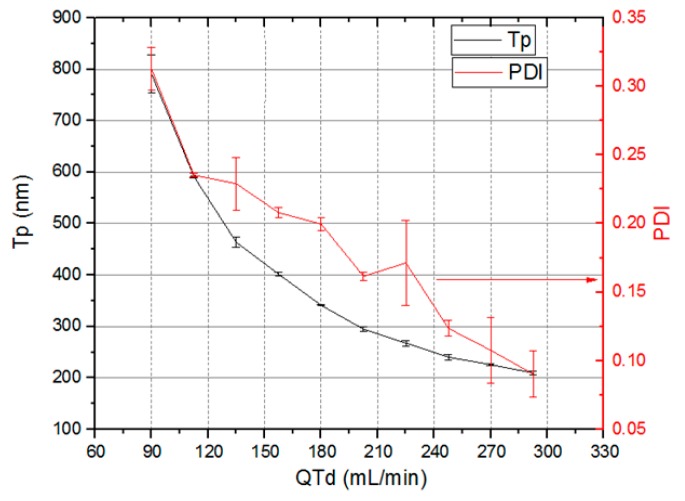
Nanocapsules sizes Tp and polydispersity index (PDI) versus total flow rate through devices.

Figure 21 shows the nanocapsules sizes (Tp), (left *Y* axis) and PDI variation (right *Y* axis) as the total system flow rate (*X* axis) varies from 90 mL/min to 293 mL/min. The total system flow rate was defined as the sum of the total emulsion flow rate and the dilution flow rate. Nanocapsule size control by means of total flow rate was achieved. The nanocapsules reduce their sizes as total flow rate through devices increase. Nanocapsules with sizes varying from 790.5 nm ± 36.9 nm to 209.7 nm ± 3.5 nm and PDI between 0.313 ± 0.016 and 0.09 ± 0.017 were obtained.

A flow rate increase in a 3D Serpentine micromixer leads to chaotic advection intensification. As a result, there is an increase in the flow streamlines crossing leading to an increase in the shearing forces. As consequence, there is a tendency to reduce the generated drops sizes in the emulsification stage. The same applies to the dilution stage, where the chaotic advection intensification lead to a better mixing between the emulsion and the dilution liquid, propitiating a higher solvent diffusion rate from inside the emulsion drops to the surrounding liquid.

The system working at total flow rate as high as 323 mL/min produced nanocapsules with a size of 193 nm and PDI of 0.109. This flow rate value is an order of magnitude higher than that previously reported in the scientific literature [80,81,82,83,84,85,86,87,88,89]. Higher flow rates were not tested, but the system is not limited to the previously mentioned value. Reducing nanocapsule size as total system flow rate increases is an advantage since it increases production rate.

We have used in our LTCC microfluidic devices internal numbering-up which consists of assembling several microreactors to obtain macroscopic flows. To increase even further the production rate it is possible to use external numbering-up which consists in assembling several LTCC microreactors in parallel. Our current research is directed to the study of external numbering-up using multilayer ceramic microreactors.

**Figure 22:**
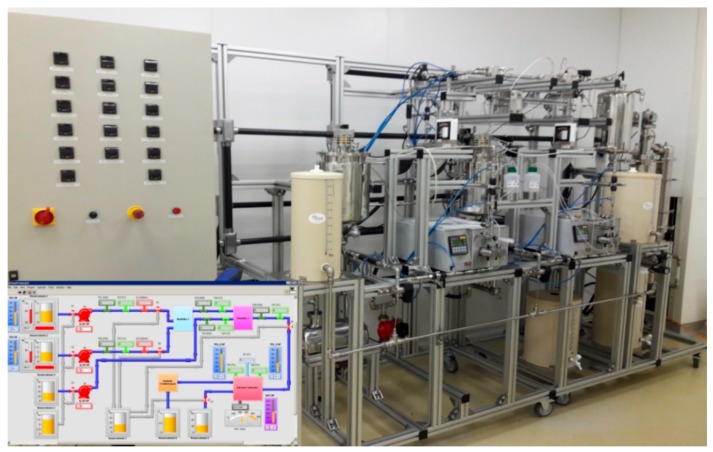
Implemented prototype for Nanocapsules production using the LTCC microfluidic devices.

We have designed and fabricated a prototype system bench for process intensification using external numbering-up of LTCC microreactors as depicted in Figure 22.

The integrated chemical synthesis and nanoparticle manufacturing system developed is a modular, continuous flow bench for chemical synthesis procedures as well as polymer nanoparticles manufacturing by the emulsification, diffusion and solvent extraction method. The system is computer controlled by using a homemade Labview virtual instrument. This bench consists of three main subsystems:

(a) Fluid management subsystem: The purpose of the fluid management subsystem is to control pressure, flow and temperature of three fluids (organic phase, aqueous phase, dilution or cleaning phase) in order to perform the desired function.
(b) Microfluidics Subsystem: The microfluidic subsystem aims to define the behavior of the system (chemical synthesis or manufacturing of nanoparticles) from LTCC microfluidic devices placed in series and parallel in frames designed for this.
(c) Solvent extraction subsystem: The solvent extraction subsystem aims to remove solvents from the fluids resulting from the previous operations and provide a suspension with solvent-free nanoparticles. The method used for this operation does not degrade the nanoparticles.

## 4. Conclusions

LTCC green tape technology is an appropriate tool to produce 3D microfluidic structures with different geometries that can implement chemical processes in severe environments.

Regarding LTCC technology applied to Chemical Processes Miniaturization, there is a long road ahead and results obtained so far stimulate new developments. In respect to Process Intensification we are aligned with the strategic guidelines proposed by Keil in [90].

We manufactured diverse LTCC microfluidic devices to carry out various chemical processes in order to fabricate micro- and nanoparticles and other unit operations. The implemented Microfluidic devices intended for micro- and nanoparticles production can operate at higher flow rates compared to common microfluidic devices, this fact helped to accomplish process numbering-up.

A Flow Focusing Device in 3D configuration (3D-FFD) was used to produce sodium alginate microparticles by ionic gelation process and Benzophenone-3 (BZ-3) submicron particles by nanoprecipitation. Sodium alginate microparticles produced with a flow rate of 3 mL/min yield particles ranging from 400 µm to 115 µm with polydispersity less than 1.5%. The nanoprecipitation experiment flow rate ratio was varied from 10 to 50 and flow rate values of 1 mL/min and 0.5 mL/min. BZ-3 particles yield in a range varying from 210 nm up to 850 nm.

A vortex micromixer was used to produce a water-in-diesel emulsion with a water/diesel ratio of 5:95. Surfactant ratio of 10% (*v*/*v*) with diesel flow rate varied from 8 mL/min to 20 mL/min renders a stable solution with water droplets with smaller sizes, in a range below 200 nm. The sizes depend on flow rate and stability varies with the surfactant concentration.

A 3D flow focalization device was used for partial solvent extraction. The simulated mean extraction efficiency yields values as high as 80.6%. In experimental results with a total flow rate of 1 mL/min, extraction efficiency of 80.8% ± 2.2% was obtained.

A microfluidic device based on a 3D serpentine micromixer was used for synthesis and functionalization of gold nanoparticles. The reaction time was reduced from 30 min (usual batch conditions) to about 1.5 min. Experimental results indicate that it is possible to vary the gold nanoparticles sizes by modulating operation parameters with reagent concentration, flow rate ratio, flow rate, and so on. Gold particles in the order of 3 to 35 nm were obtained by changing these parameters.

Finally we presented a continuous regime microfluidic system for generation of nanocapsules for pharmaceutical applications integrated by three modules: emulsification, dilution, and solvent extraction. The emulsification and dilution microfluidic devices, manufactured with LTCC technology, make use of microfluidic topologies based on 3D serpentine micromixers. The developed system generates nanocapsules with mean sizes between 790.5 nm ± 4.7% and 209.7 nm ± 1.7%, with PDI between 0.313 ± 5.1% and 0.09 ± 18.9%, with a total system flow rate that varies between 90 mL/min and 293 mL/min. This system has the potential to generate nanocapsules with narrow size distribution and control over size, operating in a continuous regime at a flow rate in the order of 320 mL/min or higher, which has not been previously reported by scientific literature.

Chemical process intensification using miniaturization technology delivers processes for continuous micro- and nanoparticle fabrication with high yield and low processing time, and is safer and environmentally friendly. Manufacturing costs of devices are low and they guarantee high chemical compatibility and low reagent consumption.

## Figures and Tables

**Table 1:** Materials.

Material	Chemical Abstracts Service (CAS)	Supplier	Used in Section	Process/Microfluidic Device
951P2 and 951PX (LTCC ceramic tapes)		DuPont	3.1; 3.2; 3.3; 3.4; 3.5	All Devices
Ultra-pure Water		Obtained in our Lab	3.1; 3.2; 3.3; 3.4; 3.5	All Devices
Sodium Alginate	9005-38-3	Sigma Aldrich	3.1.1	Ionic Gelation for Microcapsules Production/3D Flow Focusing
Mineral Oil	8042-47-5			
Calcium Chloride	10043-52-4			
Benzophenone	119-61-9		3.1.2	Nanoprecipitation/3D Flow Focusing
Isopropyl Alcohol (IPA)	67-63-0			
Methanol (MTH)	67-56-1			
Ethanol	64-17-5		3.2	Water-in-Diesel Nanoemulsion/Vortex Micromixer
Nonyphenol-Ethoxylated	68412-54-4	LGC Group Standards		
Diesel	68334-30-5	PETROBRAS		
Acetone	67-64-1	Sigma Aldrich	3.3	Solvent Extraction/3D Flow Focalization
Sodium Citrate Dehydrate	6132-04-3		3.4	Synthesis of Gold Nanoparticles
tetrachloroauric (III) Acid	27988-77-8			
Tween^®^20	9005-64-5		3.5	Emulsion–Diffusion Solvent Extraction Process/Micromixer for Nanoemulsion Generator; Micromixer for Solvent Diffusion
Ethyl Acetate	141-78-6			
Polycaprolactone (PCL) 10 kDa	24980-41-4			
Pluronic^®^F127 NF	9003-11-6			
Hydrocortisone Acetate	50-03-3			
(Caprylic/Capric Triglycerid) Miglyol^®^810 oil	65381-09-01	Mapric		

**Table 2:** Equipment.

Equipment/Model	Manufacturer	Used in Section	Purpose
Milli-Q System	Millipore Corporation, USA	All Sections	Ultra-Pure Water Production
Syringe Pump/phd 4400	Harvard Apparatus	3.1; 3.2; 3.3; 3.4	Low Pressure Pumping System
High Pressure Liquid Cromatography (HPLC) pumps/p25sfx01	Scientific Systems	3.5	High Pressure Pumping System
Micro Annular Gear Pump/MZR-2905	HNP Mikrosysteme GmbH	3.5	
Laser Diffraction Particle Analyzer/ls230	Beckman Coulter	3.1.1	Laser Diffraction Technique
Digital Microscope/vhx-6000	Keyence, USA	3.1.1	Morphological Evaluation
Scanning Electron Microscopy-Field Emission Gun (SEM-FEG)/Quanta 3d	Thermo Fisher Scientific	3.1.2	
Delsa Nano	Beckman Coulter	3.1.2	Dynamic Light Scattering (DLS) Technique
Gas Chromatograph/GC-2010 Plus	Shimadzu	3.3	Solvent Concentration Measurement
S2 Picofox	Bruker	3.4	Total Reflection X ray Fluorescence Spectroscopy (TXRF) Technique
Spectrophotometer/HP8453	Agilent	3.4	Electronic Spectroscopy (UVVis) Technique
Zetasizer Nano-ZS	Malvern	3.4; 3.5	Dynamic Light Scattering (DLS) Technique

**Table 3:** Factorial experimental planning for simulations experiences.

Number	Process Variables	Experimental Conditions		
		−1	0	1
1-	Flow Rate Ratio R_Q_	5	7,5	10
2-	Total Flow Rate *Q*_T_ (×10^−8^ m^3^/s)	1.67	9.17	16.67
3-	Main Channel Length *L*_CD_ (mm)	7.4	14.4	21.4
4-	Output Hydraulic Diameter *OD*_H_ (µm)	214.63	276.32	338
